# Management of Maternal Cardiac Arrest in the Third Trimester of Pregnancy: A Simulation-Based Pilot Study

**DOI:** 10.1155/2016/5283765

**Published:** 2016-07-31

**Authors:** Jacquelyn Adams, Jose R. Cepeda Brito, Lauren Baker, Patrick G. Hughes, M. David Gothard, Michele L. McCarroll, Jocelyn Davis, Angela Silber, Rami A. Ahmed

**Affiliations:** ^1^Summa Health System, Department of Obstetrics and Gynecology, Akron, OH 44304, USA; ^2^Summa Health System, Department of Medical Education, Virtual Care Medical Simulation Laboratory, Akron, OH 44304, USA; ^3^Biostats Inc., Canton, OH 44730, USA; ^4^Summa Health System, Summa Center for Women's Health Research, Akron, OH 44304, USA; ^5^Summa Health System, Department of Nursing Professional Development, Akron, OH 44304, USA; ^6^Summa Health System, Department of Obstetrics and Gynecology and Department of Maternal Fetal Medicine, Akron, OH 44304, USA

## Abstract

*Objective*. To evaluate confidence, knowledge, and competence after a simulation-based curriculum on maternal cardiac arrest in an Obstetrics & Gynecologic (OBGYN) residency program.* Methods*. Four simulations with structured debriefing focusing on high yield causes and management of maternal cardiac arrest were executed. Pre- and post-individual knowledge tests (KT) and confidence surveys (CS) were collected along with group scores of critical performance steps evaluated by content experts for the first and final simulations.* Results*. Significant differences were noted in individual KT scores (pre: 58.9 ± 8.9 versus post: 72.8 ± 6.1, *p* = 0.01) and CS total scores (pre: 22.2 ± 6.4 versus post: 29.9 ± 3.4, *p* = 0.007). Significant differences were noted in airway management, *p* = 0.008; appropriate cycles of drug/shock-CPR, *p* = 0.008; left uterine displacement, *p* = 0.008; and identifying causes of cardiac arrest, *p* = 0.008. Nonsignificant differences were noted for administration of appropriate drugs/doses, *p* = 0.074; chest compressions, *p* = 0.074; bag-mask ventilation before intubation, *p* = 0.074; and return of spontaneous circulation identification, *p* = 0.074. Groups remained noncompetent in team leader tasks and considering therapeutic hypothermia.* Conclusion*. This study demonstrated improved OBGYN resident knowledge, confidence, and competence in the management of third trimester maternal cardiac arrest. Several skills, however, will likely require more longitudinal curricular exposure and training to develop and maintain proficiency.

## 1. Introduction

Cardiac arrest is a rare event during pregnancy [1]. Due to advances in assisted reproductive therapy, the increasing number of pregnancies in women older than thirty-five years of age, and continued intimate partner violence globally [2], the likelihood of maternal cardiac arrest is escalating [3]. Whether cardiac arrest in pregnancy is ultimately attributed to obstetric or nonobstetric etiologies, the cause must be identified in a timely fashion and aggressively managed to improve the likelihood of successful resuscitation of both the mother and fetus. Previous research has shown simulation to be a valuable tool as a means to educate novice providers in the management of emergent obstetrical conditions [4].

Simulation has also been shown to improve competence in obstetric emergency decision-making, leadership, and individual and team performance [5]. This sentiment is reinforced in Committee Opinion Number 590 from The American College of Obstetricians and Gynecologists which states that one parameter for preparedness is integration of standardized emergency simulation and drills to identify and correct common clinical errors, to practice effective communication in a crisis, and to reinforce protocols, activation criteria, and critical interventions [6].

The objective of this study was to improve the knowledge, competence, confidence, and crisis resource management (CRM) skills of a team of obstetrical residents in the management of maternal cardiopulmonary arrest through the implementation of a simulation-based curriculum.

## 2. Methods 

### 2.1. Participants and Data Collection

Nine residents from our institution's Obstetrics & Gynecology (OBGYN) residency program were recruited for the study based on their availability to participate in all four simulations. Residents were evenly assigned to one of two study groups based on their level of training. The residents not part of the study were involved in the educational experience as part of a nonresearch group. Simulations were scheduled during protected time for weekly resident education. The study was considered exempt by the Institutional Review Board.

Participants completed a 7-question confidence survey (CS) utilizing a 5-point Likert scale and a 20-question multiple-choice knowledge test (KT) in a pretest/posttest approach. Competence was assessed by video review of obstetrical residents' management as a team of a simulated maternal cardiac arrest in a similar pretest/posttest fashion. An expert panel composed of a Maternal Fetal Medicine (MFM) attending, an Emergency Medicine (EM) attending, and an Obstetrician/Gynecologist graded the pre- and postintervention simulations using a modified score sheet with items from both TeamSTEPPS® and the American Heart Association® (AHA) Megacode Checklist (see the following checklist).

*Resuscitation Checklist for the Management Cardiac Arrest in Pregnancy* (Modified from the AHA Megacode Testing Checklist 3)

*Critical Performance Steps*

*Team Leader (Check if Done Correctly)*

□ Ensures high-quality CPR at all times.
□ Assigns team member roles.
□ Ensures that team members perform well.

*VF Management (Check if Done Correctly)*

□ Recognizes VF.
□ Clears before ANALYZE and SHOCK.
□ Immediately resumes CPR after shocks.
□ Appropriate airway management.
□ Appropriate cycles of drug-rhythm check/shock-CPR.
□ Administers appropriate drug(s) and doses.

*BLS/ACLS Modifications for Pregnancy (Check if Done Correctly)*

□ Activates protocol for an emergency cesarean delivery as soon as cardiac arrest is identified.
□ Performs manual left uterine displacement.
□ Performs chest compressions slightly higher on the sternum than normally recommended.
□ Uses bag-mask ventilation with 100% O_2_ before intubation is done.
□ Attempts to identify common and reversible causes of cardiac arrest in pregnancy.
□ Delivers infant by emergency cesarean section.
□ Delivers infant no more than 5 minutes after cardiac arrest ensues.

*Postcardiac Arrest (Check if Done Correctly)*

□ Identifies ROSC.
□ Ensures BP and 12-lead ECG are performed and O_2_ saturation is monitored, verbalizes need for endotracheal intubation and waveform capnography, and orders laboratory tests.
□ Considers therapeutic hypothermia.

### 2.2. Curriculum Design

**Figure 1:**
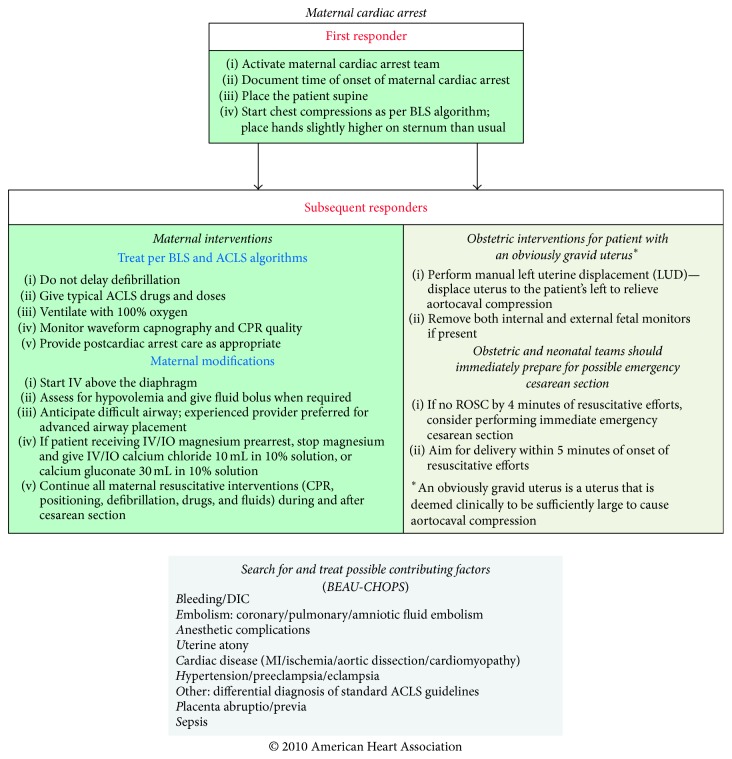
Maternal cardiac arrest algorithm.

The medical simulation staff, two senior level OBGYN residents, and the Director of the MFM department at our institution jointly designed a four-case simulation based curriculum. Educational objectives were modeled after the current AHA guidelines for the management of maternal cardiac arrest (see Figure 1) and the TeamSTEPPS CRM course [7, 8].

All simulations were based on presentations that could lead to maternal cardiac arrest [9]. The four scenario stems were massive pulmonary embolism, magnesium toxicity, placental abruption due to motor vehicle accident, and blunt abdominal trauma caused by intimate partner violence. Data collection was performed prior to the first and final simulations. This data collection process included a confidence survey, knowledge test, and evaluation of the team simulation scenarios by the faculty content experts. No evaluation was performed for the intervention phase simulation scenarios. During the intervention phase residents managed two additional cases utilizing a deliberate practice approach [10]. All four simulations were followed by faculty led debriefing on team performance by our panel of content experts. The knowledge test and confidence surveys were not collected for the intervention phase. All data collected was prior to and following the intervention phase. Videos were reviewed and scored by content experts/faculty of the first summative simulation and the final (4th) summative simulation.

### 2.3. Materials and Models

All simulations were performed in the simulation laboratory of Summa Health System. A confederate nurse and real time patient feedback were used in all simulations to facilitate information gathering and scenario flow. A digital monitor displaying dynamic vital signs was readily available and modified based on case progression. Cases were ended at the faculty member's discretion once 10 minutes after maternal cardiac arrest had elapsed.

**Figure 2:**
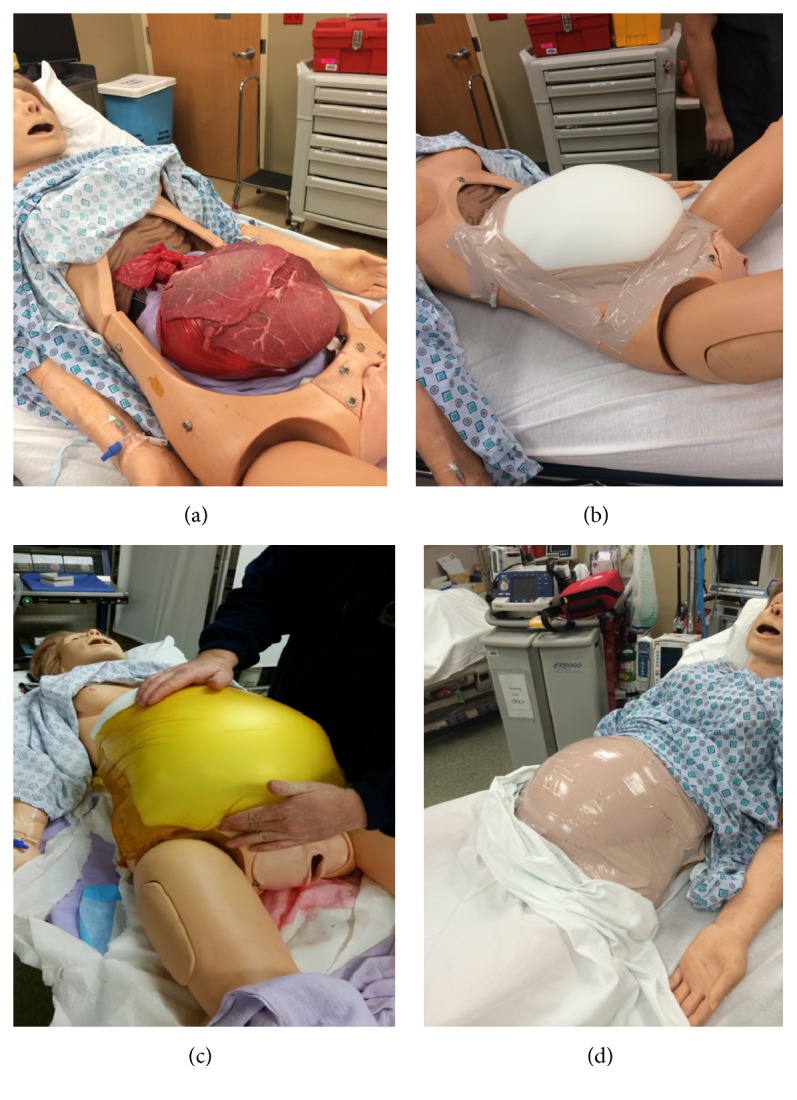
Modified Gaumard's NOELLE® Maternal and Neonatal Birthing Simulator. (a) Disposable abdominal wall and amniotic sac unit covered by flank steak, emulating fascia, and muscle layers. (b) Subcutaneous fat later made from two-inch model foam. (c) Internal layers held in place by an iodoform band. (d) Skin recreated from flesh-colored duct tape.

Gaumard's NOELLE Maternal and Neonatal Birthing Simulator was used for all simulations. To enable the performance of an emergent perimortem cesarean section, the simulator was fitted with a disposable abdominal wall and amniotic sac unit. The amniotic sac was emulated by a red biohazard bag, the NOELLE infant model (basic), water, and food coloring. The simulator electrical and mechanical systems were deactivated and protected by plastic liners. The amniotic sac unit was placed in the abdomen of the NOELLE simulator. This unit was then covered by flank steak to simulate the fascia and muscle layers of the abdomen. These two layers were then protected by two-inch model foam to serve as the subcutaneous fat. To hold the layers of the abdomen in place, an iodoform band was attached from the pelvis to the breast line of the model. The skin was recreated by flesh-colored duct tape keeping the abdominal layers secure (see Figure 2). A standard crash cesarean section kit was available upon request from the resuscitation team to allow for performance of emergent perimortem cesarean section.

### 2.4. Data Analysis

Individual CS, KT, and simulation team performance scores were analyzed using SPSS 22.0.

## 3. Results 

Resident participation was 45% (9 of 20). Average participant age was 29.6 ± 1.1 years (*n* = 9). Significant differences were noted in individual KT scores (pre: 58.9 ± 8.9 versus post: 72.8 ± 6.1, *p* = 0.01) and CS total scores (pre: 22.2 ± 6.4 versus post: 29.9 ± 3.4, *p* = 0.007). Significant differences were noted in group competencies of airway management, *p* = 0.008; appropriate cycles of drug shock-CPR, *p* = 0.008; left uterine displacement, *p* = 0.008; and identifying causes of cardiac arrest in pregnancy, *p* = 0.008. Nonsignificant differences were noted after intervention on the residents' competencies for administration of appropriate drugs/doses, *p* = 0.074; chest compressions, *p* = 0.074; bag-mask ventilation before intubation, *p* = 0.074; and identification of return of spontaneous circulation identification, *p* = 0.074. Groups remained noncompetent in providing high-quality cardiopulmonary resuscitation at all times, team leader assigning roles, or considering therapeutic hypothermia. See Tables 1 and 2 for results.

## 4. Discussion 

In our study we noted a significant improvement in the critical performance steps of airway management, adherence to advanced cardiovascular life support (ACLS) treatment algorithm, manual left uterine displacement, and identification of common causes of maternal cardiac arrest. There was also improvement of KT scores from baseline. This trend was also demonstrated in the increase in CS from the initial evaluation to the postintervention stage. This is likely a result of the focused, interactive learning environment afforded in the simulation laboratory and the expert debriefing following all simulations.

Cardiopulmonary arrest in pregnancy is a high-risk, low frequency scenario and as such requires continued training and preparation to maintain an appropriate skill level to manage these complex patients [11–13]. The use of simulation to improve outcomes has been demonstrated in multiple arenas because it gives the learner the opportunity to experience uncommon scenarios, receive feedback, and perhaps most importantly correct mistakes before they become part of the learner's or group's mental model [14].

Despite the training intervention, groups did not demonstrate a significant improvement in team leader specific tasks of ensuring high-quality cardiopulmonary resuscitation (e.g., minimizing disruptions in chest compression) and role assignment. During the debriefing sessions team leaders expressed difficulty assigning roles and determining a team leader because, in some instances, there were multiple senior level residents in the teams. This varies greatly from their normal call schedule. We used this opportunity to emphasize the importance of having a clearly defined leader, how to choose a team leader among peers, and how those “would be leaders” can be an asset to the team by being the first follower [15]. We also emphasized the importance of closed-loop communication during the resuscitation to improve team communication and avoid management errors [16].

The critical performance steps that did not show improvement, pertaining to CRM (e.g., team leadership), likely need continued longitudinal training throughout residency to achieve competence. Consistent with current ACLS training literature regarding skill decay, our belief is that the lack of consideration of therapeutic hypothermia, as well as the areas where residents remained noncompetent, was likely due to the expected decline in knowledge and psychomotor skill retention after ACLS certification [17].

**Figure 3:**
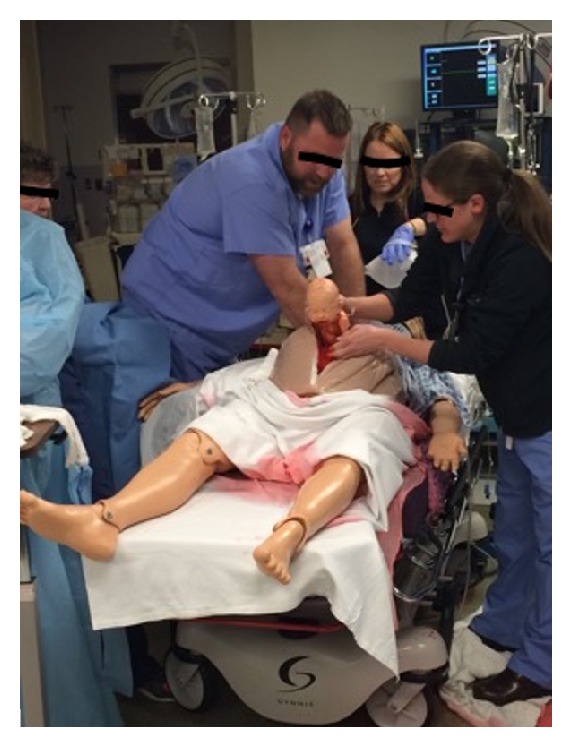
Residents performing emergent perimortem cesarean section.

Residents performed a perimortem cesarean section during all simulations. However, we observed an unexpected trend with almost all groups choosing to perform a Pfannenstiel skin incision and a low transverse uterine incision, contrasting with the current practice guidelines that favor a midline vertical incision (see Figure 3). Residents reported feeling they would be more proficient and could expedite delivery using this approach. This decision-making process has been supported in the literature by other authors [18, 19]. Group debriefing highlighted the advantages of a midline abdominal incision particularly for cases of abdominal trauma, where general surgery may need to be involved in the resuscitative efforts.

Our study had several limitations including a small sample size and residents from one concentration (obstetrics and gynecology) from a single institution. We hope to further strengthen our simulation-based curriculum by providing a true interprofessional learning environment. In future simulation scenarios, ideally we would include multiple disciplines and levels of training to help the assignment of team roles and execution of tasks feel more appropriate for the learner. Based on our study, further practice with these scenarios is needed to solidify the important concepts pertaining to cardiovascular collapse. In our institution, we plan to do this by utilizing these scenarios starting with the incoming interns' initial “boot camp” and repeating this training through all four years to track progress, decrease skill decay, and solidify concepts with which the learner may not be regularly faced. For other groups attempting a similar study or for obstetrics and gynecology learners in general, our project demonstrated the need to enforce early and often the key changes for maternal cardiac arrest management. It also demonstrated that further training and enforcement of team leader skills is crucial to improve the confidence and performance of the team.

## 5. Conclusion 

This simulation-based study demonstrated improved OBGYN resident knowledge, confidence, and competence in several areas of the management of third trimester maternal cardiac arrest. Several skills, however, will likely require more longitudinal curricular exposure and training to develop and maintain proficiency.

## Figures and Tables

**Table 1:** Residents confidence and knowledge scores before and after intervention.

Test scores	Before intervention	After intervention	Change in score	*p* value^*∗*^
Knowledge (%)				
Mean (SD)	58.9 (8.94)	72.8 (6.18)	13.9 (11.93)	0.016
Median	65	75	10	
Min–Max	45–70	65–80	0–35	

Self-reported confidence				
Mean (SD)	22.2 (6.42)	29.9 (3.41)	7.7 (4.82)	0.007
Median	24	32	7	

Note: ^*∗*^ *p* value via Wilcoxon signed rank test.

**Table 2:** Residents team competence scores (paired) before and after intervention.

Critical performance steps (area of competency/posttest competency)		Pretest competency		
		Not competent	Competent^∧^	*p* value^&^
Team leader				
Ensures high-quality CPR at all times	Not Competent	9 (100%)		NA
	Competent			
Assigns team member roles	Not Competent	9 (100%)		NA
	Competent			
Ensures that team members perform well	Not Competent	5 (55.6%)		0.134
	Competent	4 (44.4%)		

VF management				
Recognizes VF	Not Competent	5 (55.6%)		0.134
	Competent	4 (44.4%)		
Clears before ANALYZE and SHOCK	Not Competent			NA
	Competent		9 (100%)	
Immediately resumes CPR after shocks	Not Competent			NA
	Competent		9 (100%)	
Appropriate airway management	Not Competent			0.008
	Competent	9 (100%)		
Appropriate cycles of drug-rhythm check/shock-CPR	Not Competent			0.008
	Competent	9 (100%)		
Administer appropriate drug(s) and doses	Not Competent	9 (100%)		NA
	Competent			

BLS/ACLS modifications for pregnancy				
Activates protocol for an emergency cesarean delivery as soon as cardiac arrest is identified	Not Competent			0.134
	Competent	4 (44.4%)	5 (55.6%)	
Positions patient in left-lateral tilt or performs manual uterine displacement	Not Competent			0.008
	Competent	9 (100%)		
Performs chest compressions slightly higher on sternum than normally recommended	Not Competent			0.074
	Competent	5 (55.6%)	4 (44.4%)	
Uses bag-mask ventilation with 100% O_2_ before intubation is done	Not Competent			0.074
	Competent	5 (55.6%)	4 (44.4%)	
Attempts to identify common and reversible causes of cardiac arrest in pregnancy	Not Competent			0.008
	Competent	9 (100%)		
Delivers infant by emergency cesarean section	Not Competent			NA
	Competent		9 (100%)	
Delivers infant no more than 5 minutes after cardiac arrest ensues	Not Competent			0.074
	Competent	5 (55.6%)	4 (44.4%)	

Postcardiac arrest				
Identifies ROSC	Not Competent			0.074
	Competent	5 (55.6%)	4 (44.4%)	
Ensures BP and 12 lead ECG are performed and O_2_ saturation is monitored, verbalizes need for endotracheal intubation and waveform capnography, and orders laboratory test	Not Competent	9 (100%)		NA
	Competent			
Considers therapeutic hypothermia	Not Competent	9 (100%)		NA
	Competent			

Note: ^&^ *p* value via McNemar's test. ^∧^Competency is defined by a majority of rater's competency determination.

## Abbreviations

CRM: Crisis resource management
OBGYN: Obstetrician/Gynecologist
CS: Confidence survey
KT: Knowledge test
MFM: Maternal Fetal Medicine
EM: Emergency Medicine
AHA: American Heart Association
CPR: Cardiopulmonary resuscitation
ACLS: Advanced Cardiac Life Support.
